# MicroRNA-486-3p Regulates γ-Globin Expression in Human Erythroid Cells by Directly Modulating BCL11A

**DOI:** 10.1371/journal.pone.0060436

**Published:** 2013-04-04

**Authors:** Valentina Lulli, Paolo Romania, Ornella Morsilli, Paolo Cianciulli, Marco Gabbianelli, Ugo Testa, Alessandro Giuliani, Giovanna Marziali

**Affiliations:** 1 Department of Hematology, Oncology and Molecular Medicine, Istituto Superiore di Sanità, Rome, Italy; 2 Paediatric Haematology/Oncology Department, Bambino Gesù Children’s Hospital IRCCS, Rome, Italy; 3 Thalassemia Unit, “Sant’ Eugenio” Hospital, Rome, Italy; 4 Department of Environment and Health, Istituto Superiore di Sanità, Rome, Italy; University of California, San Francisco, United States of America

## Abstract

MicroRNAs (miRNAs) play key roles in modulating a variety of cellular processes through repression of mRNAs target. The functional relevance of microRNAs has been proven in normal and malignant hematopoiesis. While analyzing miRNAs expression profile in unilineage serum-free liquid suspension unilineage cultures of peripheral blood CD34^+^ hematopoietic progenitor cells (HPCs) through the erythroid, megakaryocytic, granulocytic and monocytic pathways, we identified miR-486-3p as mainly expressed within the erythroid lineage. We showed that miR-486-3p regulates BCL11A expression by binding to the extra-long isoform of BCL11A 3′UTR. Overexpression of miR-486-3p in erythroid cells resulted in reduced BCL11A protein levels, associated to increased expression of γ-globin gene, whereas inhibition of physiological miR-486-3p levels increased BCL11A and, consequently, reduced γ-globin expression. Thus, miR-486-3p regulating BCL11A expression might contributes to fetal hemoglobin (HbF) modulation and arise the question as to what extent this miRNA might contribute to different HbF levels observed among β-thalassemia patients. Erythroid cells, differentiated from PB CD34^+^ cells of a small cohort of patients affected by major or intermedia β-thalassemia, showed miR-486-3p levels significantly higher than those observed in normal counterpart. Importantly, in these patients, miR-486-3p expression correlates with increased HbF synthesis. Thus, our data indicate that miR-486-3p might contribute to different HbF levels observed among thalassemic patients and, possibly, to the clinical severity of the disease.

## Introduction

MicroRNAs (miRNAs) are a class of naturally occurring, small non-coding RNA molecules, about 21–25 nucleotides in length and highly conserved during evolution [1]. They are potent negative regulators of gene expression modulating the translation of hundreds of genes by binding to cognate sites in the 3′ untranslated region (UTR) of target mRNAs [2], [3]. Depending on the degree of sequence complimentary miRNA binding will result in the inhibition of translation and/or degradation of target mRNAs [4]. The complexity of translation inhibition can be further extended through heterotypic miRNA-mRNA interactions, as genes can harbor binding sites for several miRNAs. Thus, although their inhibitory effects on individual mRNAs are generally modest, their combined effects on multiple mRNAs can evoke strong biological responses.

miRNAs play a powerful role in hematopoiesis, where they have been implicated in cell fate specification, proliferation, differentiation, etiology and progression of cancer [5], [6]. Alteration of specific miRNAs expression can produce dramatic phenotypes leading to severe hematopoietic defects. On the contrary, guided modulation of miRNAs expression have been suggested as a novel approach to develop innovative therapeutic protocols.

Several miRNAs were identified as putatively critical for erythroid development and maturation [7], [8]. Erythropoiesis was reported to be promoted by miR-451 and miR-144 [9], [10] and negatively regulated by miR-150 [11], miR-221, miR-222 [12] and miR-223 [13]. MiR-15b, miR-16, miR-22, and miR-185 were found to have strong positive correlation with the appearance of erythroid surface antigens and hemoglobin synthesis [14], [7], [8].

Several miRNAs have been implicated in the developmental progression of globin gene expression and, particularly, in the reactivation of γ-globin gene expression associated with increased fetal hemoglobin (HbF) synthesis [7]. In adults, variable levels of HbF may persist without clinical consequence; rather high levels of HbF have a major impact in ameliorating the severity of the principal hemoglobin disorders, such as sickle cell anemia and β-thalassemia [15]. The degree of HbF persistence varies greatly between adult individuals and this variability is at a large extent genetically controlled [16].

The principle that elevated HbF ameliorates the severity of the β-hemoglobin disorders has been the driving force behind efforts to stimulate fetal hemoglobin production during the last twenty years.

Elevated miR-210 levels have been observed in the context of elevated γ-globin levels in two cases of hereditary persistence of HbF [17], while the let-7 family has been associated with hemoglobin switching [18]. Further, two miRNAs, miR-221 and miR-222, have been identified to regulate HbF expression in erythropoietic cells via modulation of the kit receptor [19].

Recently, it has been reported that increased expression of miR-15a and miR-16-1 in human erythroid cells results in high fetal and embryonic hemoglobin gene expression [20]. This effect is mediated, at least in part, through down-modulation of MYB, an inhibitor of the γ-globin gene transcription. Further, miR-96 was identified as a regulator of HbF expression by direct post-transcriptional inhibition of γ-globin mRNA during adult erythropoiesis [21].

Recent genetic studies focused on natural variation of HbF expression level in human population established BCL11A as a new regulator of both developmental control of hemoglobin switching and silencing of γ-globin expression in adults [22], [23]. BCL11A is a highly conserved zinc finger protein essential for normal B and T cell development and is involved in lymphoid malignancies through translocation or amplification events; it is expressed in hematopoietic progenitors and down-regulated during myeloid differentiation [24], [25]. Lentiviral vector mediated knockdown of BCL11A in erythroid cells was associated with increased levels of γ-globin and HbF production, without affecting erythroid differentiation. This indicates that BCL11A modulates HbF levels via direct transcriptional repression of the γ-globin gene, rather than through alteration of erythroid kinetics, as suggested for the HBS1L-MYB locus [26], [27], [28], [29]. The exact mechanism by which BCL11A silences γ-globin expression remains still unclear. A recent study suggests that this may be mediated through both interactions with transcription factors, such as SOX6, that binds chromatin at the proximal γ-globin promoters and through long-range interactions with a variety of regions throughout the γ-globin gene cluster [30].

A better understanding of the mechanisms mediating HbF production in the human adult may lead to novel therapeutic approaches for β-globin disorders.

This study focuses on the role of miR-486-3p in regulating HbF expression through modulation of BCL11A in primary adult erythroid cells. The human miR-486, located on chromosome 8p11 within the Ankyrin-1 (ANK1) gene, encodes for two mature miRNAs: miR-486-3p and miR-486-5p. In particular, we report that in the adult hematopoietic system miR-486-3p is abundantly expressed only in erythroid cells. We demonstrate that miR-486-3p directly targets BCL11A mRNA and we show that alteration of miR-486-3p expression regulates BCL11A protein levels modulating γ-globin expression. These findings indicate that miR-486-3p contributes to HbF regulation by post-transcriptional inhibition of BCL11A expression during adult erythropoiesis.

## Methods

### HPCs Purification, Erythroid Cultures

Human adult peripheral blood (PB) and cord blood (CB) were obtained after informed written consent and processed under approval of the Istituto Superiore di Sanità ethics committee. Peripheral blood (20–30 mL) of thalassemic patients was obtained after informed written consent, at Sant’Eugenio Hospital, Rome, Italy, in accordance with the Declaration of Helsinki. This study was approved by the local ethical Committee of the Sant’Eugenio Hospital. The CD34^+^ HPCs were prepared from a buffy coat preparation derived from 450 ml blood donation of healthy donors.

Blood was collected in preservative-free, citrate/phosphate/dextrose/adenine (CPDA-1) anticoagulant. A buffy coat was obtained by centrifugation (Beckman J6M/E, 1400 rpm for 20 minutes at room temperature; Beckman Instruments, Fullerton, CA). Low-density cells (less than 1.077 g/mL) were isolated using a Ficoll gradient as previously described [31], [32]. The CD34^+^ HPCs were then purified by using the MiniMACS CD34 isolation system (Miltenyi, Bergisch Gladbach, Germany), following the manufacturer’s instructions.

Unilineage cultures were performed as previously described [13], [31], [33]. Particularly, for erythroid unilineage culture purified HPCs were grown in fetal calf serum (FCS) -free liquid culture (5×10^4^ cells/mL/well) in a fully humidified atmosphere of 5% CO_2_/5% O_2_/90% N_2_ and medium was supplemented with 0.01 U/mL IL-3, 0.001 ng/mL GM-CSF (PeproTech Inc. Rocky Hill, NJ, USA) and 3 U/mL erythropoietin (Amgen Thousand Oaks, CA, USA).

For morphological analysis, cells were smeared on glass slides by cytospin centrifugation, stained with May-Grünwald-Giemsa and analyzed at 400x or 600x magnification under a microscope (Eclipse 1000, Nikon, Tokyo, Japan) equipped with a digital camera.

### γ-chain Content

High-performance liquid chromatography (HPLC) separation of globin chains was performed according to previously published method [33], [34].

### Flow Cytometry Analysis

Cells were washed and incubated 30 minutes on ice in the dark with 3–5 µg/ml PE conjugated monoclonal antibody to GPA, CD36, or isotype-matched control (Pharmingen, San Diego, CA, USA). Cells were then washed and analyzed on a FACSCanto (Becton Dickinson, Franklin Lakes, NJ, USA). Acquisition and analysis were performed using Diva software (Becton Dickinson).

### Plasmids

The human XL BCL11A 3′ UTR (NM 022893) (from 252 bps to 1858 bps) segments, containing the target site for miR-486-3p, were amplified by PCR from genomic DNA and cloned into pGL3 Control vector (Promega) in the XbaI site immediately downstream the stop codon of the luciferase gene.

The following primers were used: 5′ CTCACCGTTTGAATGCATGATCTGTATGGG 3′ and 5′ CAGTGCACTTAATTGTCCTATCTGAGCAGG 3′. The deletion of the miR-486-3p site was obtained by restriction enzyme digestion (Bgl2).

### Cell Culture and Luciferase Reporter Gene Assays

293T cells (Invitrogen, Carlsbad, CA, USA) were grown in DMEM medium supplemented with 10% fetal calf serum (FCS) and antibiotics. For luciferase assay cells were co-transfected with 0.8 µg of pGL3-3′UTR plasmid, 0.1 µg of Renilla expressing vector and 40 pmol of either a stability enhanced non-targeting dsRNA control oligonucleotide (Ambion,) or a stability-enhanced miRNA-486-3p (Ambion), all combined with Lipofectamine2000 (Invitrogen). After 48 h cells were washed and lysed according to manufacturer’s protocol (Promega, Madison, WI, USA) and luciferase activity was measured. Relative luciferase activity was obtained by normalizing the activity with the renilla luciferase.

### Erythroid Cells Transfection

PB CD34^+^ progenitors cultured in erythroid medium were transfected on day one. Cells were seeded (1,25×10^5^ cells/mL) in antibiotic-free media and transfected with 160 nM of a stability-enhanced miRNA-486-3p (Ambion), or with 160 nM of a stability enhanced non-targeting dsRNA control oligonucleotide (Ambion); for in vivo knockdown of miR-486-3p was used 160 nM of either anti miR-486-3p LNA-modified oligonucletide (Exiqon) and a LNA-modified non-targeting control miRNA (Exiqon). The LNA-modified and the stability-enhanced oligonucleotides were delivered by Lipofectamine 2000.

### RNA Extraction and Reverse Transcriptase Polymerase Chain Reaction

Total RNAs were extracted using Trizol reagent and reverse transcribed by Moloney murine Leukemia virus reverse transcriptase (Invitrogen, Carlsbad, CA, USA) with random primers.

BCL11A mRNA isoforms and γ-globin mRNA were detected by SYBR Green system and normalized with GAPDH using the following primers:

BCL11A-XL: For 5′ CAGCGGCACGGGAAGTGGAG 3′.

Rev 5′CGCCCGTGTGGCTTCTCCTG 3′.

BCL11A-L: For 5′ CAGCGGCACGGGAAGTGGAG 3′.

Rev 5′ ACGCCGAATGGGGGTGTGTG 3′.

BCL11A-S: For 5′ AGCGAACACGGAAGTCCCCTGA 3′.

Rev 5′ GGGCTCTCGAGCTTCCATCCG 3′.

BCL11A-XS: For 5′ GAGGTTGGCATCCAGGTCACG.

Rev 5′ GTACACAAATACATCCTCCAGTTCAGTC.

γ-globin [23]: For 5′ TGGATGATCTCAAGGGCAC 3′ Rev 5′ TCAGTGGTATCTGGAGGACA 3′.

GAPDH: For 5′ ACCTGACCTGCCGTCTAGAAAA 3′.

Rev 5′ CCTGCTTCACCACCTTCTTGA 3′.

Real-time PCR for miR-486-3p, miR-486-5p and miR-451 was performed using TaqMan® MiRNA Assays protocol (assay ID 002093, ID001278 and ID001141 Applied Biosystems, Foster City, CA, USA). Briefly, reverse transcriptase reaction was performed using 10 ng of total RNA and 50 nM miRNA specific stemloop RT primers. Real-time PCR was performed using standard protocol. All reactions were run in duplicate. Normalization was performed by using RNU6B primer kit (ID 001093, Applied Biosystems). Relative expression was calculated with relative standard curves for both the miRNA of interest and the endogenous control. RT-PCR analysis was performed using an ABI Prism 7900 Sequence Detector (Applied Biosystems, Foster City, CA, USA).

### Whole Cell Extracts, Western Blotting

Cells were washed and pellets resuspended in lysis buffer (20 mM HEPES, 50 mM NaCl, 10 mM EDTA, 2 mM EGTA and 0,5% (vol/vol) Nonidet P-40 supplemented with protease inhibitors), incubated for 20 min at 4°C and centrifuged for 10 min at 7000 g. Whole cell extracts were resolved by sodium dodecyl sulphate polyacrylamide gel electrophoresis, transferred onto Hybond-C paper (Amersham Biosciences), incubated with monoclonal anti-BCL11A (ABCAM), anti-γ-globin (Santa Cruz) and anti-actin (Oncogene Research, San Diego, CA, USA), as loading control, and detected using ECL detection kit (Pierce, Rockford, IL, USA). The expression levels were analyzed by the Scion Image Software (Scion, Frederich, MD, USA).

### Statistical Analysis

Data are presented as mean values and error bars indicate standard deviation (SD). The groups were compared by two-way analysis of variance (ANOVA) using Bonferroni’s test. p<0.05 was considered statistically significant. The Spearman rank correlation coefficient (r) was used to assess the correlation among miR-486-3p, BCL11A and *Η*b*F*in both direct and partial modes.

## Results

### MiR-486-3p and miR-486-5p Expression in Human Unilineage Erythroid Cultures Derived from cord- and Peripheral- Blood CD34^+^ Hematopoietic Progenitor Cells

While studying miRNA expression profiles in unilineage culture of cord blood (CB) CD34^+^ HPCs through the erythroid (E), megakaryocytic (Mk), granulocytic (G) and monocytic (Mo) lineages differentiation/maturation, we observed that miR-486 is mainly expressed in E cells and remained steadily high over time [31].

**Figure 1:**
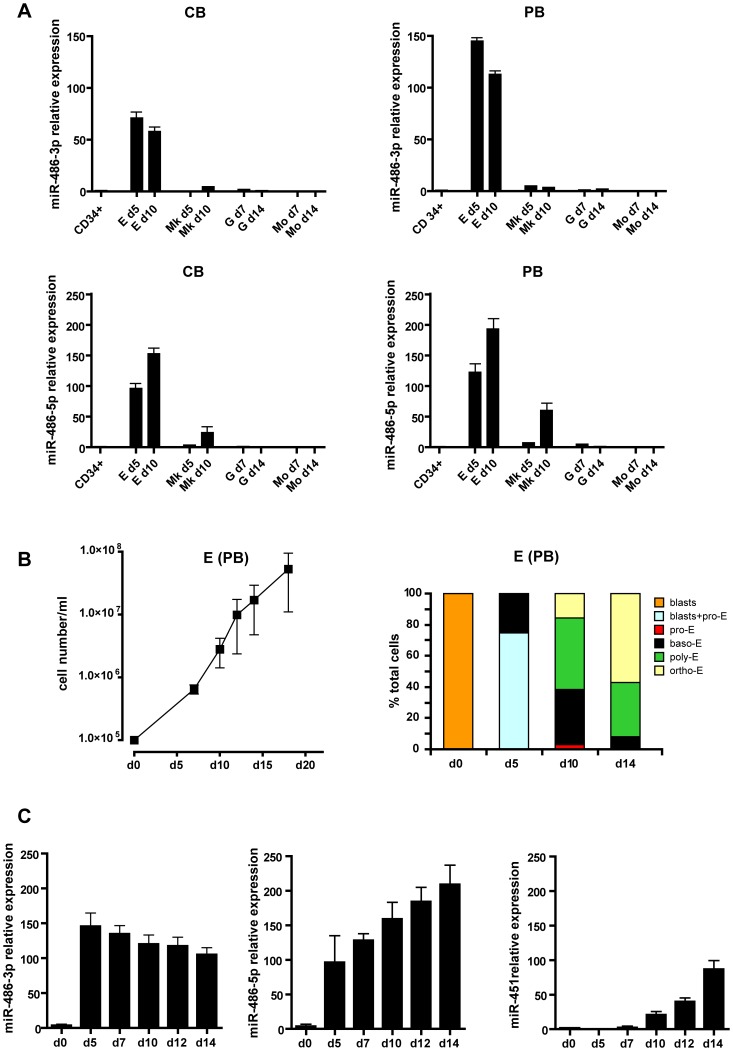
Expression of miR-486-3p and miR-486-5p in hematopoietic cultures. (**A**) Real-time PCR of miR-486-3p (upper panels) and miR-486-5p (lower panels) in erythroid (E), megakaryocytic (Mk), granulocytic (G) and monocytic (Mo) unilineage cultures derived from cord (CB) (left) and peripheral blood (PB) (right) CD34^+^ hematopoietic progenitor cells (HPCs). (**B**) Growth and differentiation of PB unilineage erythroid cultures. Percentage of blasts, differentiating proerythroblasts (pro-E), basophilic (baso-E), polychromatophilic (poly-E) and mature orthocromatic (ortho-E) erythroblasts with respect to total cells is indicated. Representative experiments are presented. Error bars represent standard deviation and indicate the average values from three independent experiments. (**C**) Expression of miR-486-3p, miR-486-5p and miR-451 in PB CD34^+^ HPCs (day 0) and at sequential days of erythroid culture. In all Real-time PCR (A, C) samples were normalized for RNU6B expression and the error bars represent the mean value ± SD (n = 3).

Peripheral blood (PB)-derived unilineage cultures might show different miRNA expression profile compared to CB [35]. To assess differences, if any, between CB and PB-derived cultures we analyzed the expression profile of miR-486-3p and miR-486-5p in unilineage E, MK, G and Mo serum-free liquid suspension cultures derived from both CB and PB CD34^+^ HPCs, respectively. Total RNA was extracted from CD34^+^ HPCs and at different days of culture, corresponding to discrete sequential stages of differentiation/maturation process, from each lineages, and analyzed by Real Time RT-PCR. As shown in Figure 1A, both miRNAs have a similar expression profile in CB and PB derived cultures. miR-486-3p and miR-486-5p are essentially undetectable in starting HPCs, whereas their level primarily and strongly increased during E differentiation. Interestingly, the expression levels of both miRNAs are higher in PB than in CB, but the relevance of this finding to normal physiology is not clear.

**Figure 2:**
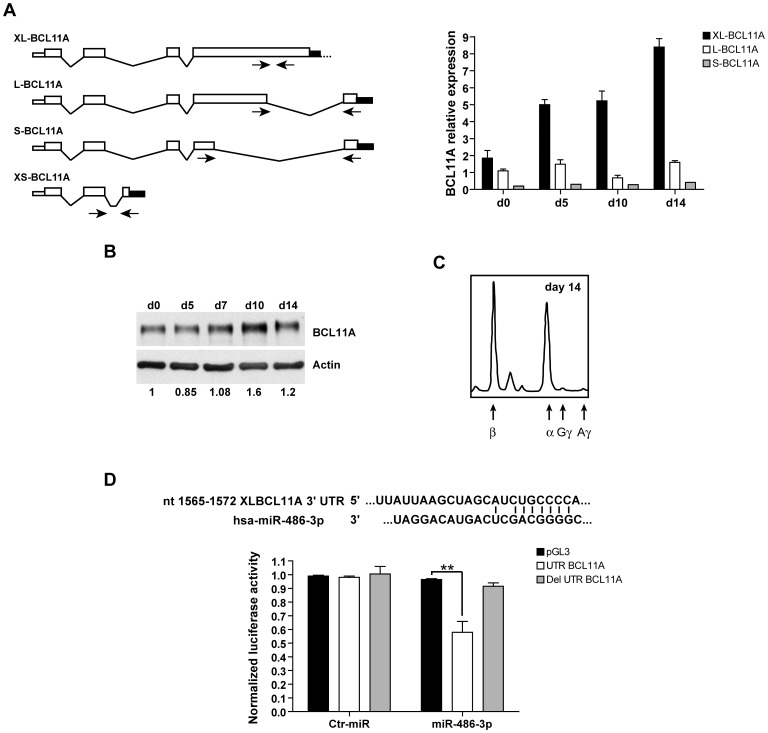
XL BCL11A isoform is a direct target of miR-486-3p. (**A**) Schematic diagram of alternative splicing of human BCL11A mRNAs. The specific primers, recognizing the different isoforms, were indicated by arrows. Relative mRNA expression of human BCL11A isoforms in PB unilineage E culture were quantified by Real-Time PCR. Transcript levels were normalized against human GAPDH transcript levels. Error bars represent standard deviation and indicate the average values from three independent experiments. (**B**) Analysis of BCL11A protein expression in erythroid culture by western blot. The XL and L bands migrate together. Values represented the ratio between BCL11A protein and actin normalized on PB CD34^+^ HPCs. A representative experiment out of three is shown. (**C**) γ-globin chain synthesis. Representative HPLC scan of globin chains synthesis observed in mature erythroblasts (day 14 of culture) derived from PB CD34^+^ HPC unilineage erythroid cultures. (**D**) BCL11A is a target of miR-486-3p. (*top*) Putative miR-486-3p binding site in the XL BCL11A 3′UTR. (*bottom*) Luciferase activity in 293T cells co-transfected with either pGL3 BCL11A XL 3′UTR wild type and miR-486-3p or non-targeting oligonucleotide (ctr-miR). As controls the pGL3 BCL11A XL 3′UTR with deleted binding site or the pGL3 control empty vector were transfected with either mimic miR-486-3p or ctr-miR. Firefly luciferase values normalized for renilla luciferase are presented. Mean ± SD from four independent experiments.

The detailed miR-486-3p and miR-486-5p expression profiles were then analyzed in PB unilineage erythroid culture. In this serum-free culture, purified HPCs are induced to selective E growth by very low dosages of IL-3, GM-CSF, and saturating erythropoietin (Epo) levels. Cell growth (Figure 1B, *left*) is associated with a gradual decrease of CD34^+^ marker during the first seven days of culture, followed by a progressively increasing expression of specific membrane markers such as glycophorin A (GPA) (data not shown) [13], [31]. Consistently, cell morphological analysis showed a gradual wave of maturation along the differentiative pathway to terminal mature erythroid cells (Figure 1B, *right*). This culture system results in robust expression of adult hemoglobin (α and β globin chains) and low level of HbF synthesis (Gγ and Aγ globin chains) (Figure 2C). Thus, this in vitro model closely reproduces the in vivo erythropoiesis pattern in terms of cell proliferation and differentiation/maturation, while allowing us to sample the cultures at different times in order to analyze sequential, discrete stages of maturation of the erythropoietic pathway in a more than 95% (96.5±1.8) pure erythroid cell population.

In these unilineage erythroid cultures miR-486-3p and miR-486-5p were strongly expressed through the differentiation process until terminal maturation (Figure 1C). To better analyze the relative abundance of miR-486-3p and miR-486-5p in erythroid cells, their levels of expression were compared to that of miR-451, well known for its high erythropoietic-restricted expression in the hematopoietic system [9], [10]. As shown in figure 1C during the initial and middle stages of erythroid differentiation (i.e. day 5 and 7), miR-486-3p and miR-486-5p display a far higher expression level than miR-451. At late differentiative stage, miR-486-5p shows the greatest expression, whereas miR-486-3p and miR-451 have more comparable expression levels, consistent to the highest expression of miR-451 in mature erythroid cells. It is interesting to note that there is a remarkable difference in the expression pattern of miR-486-5p and miR-486-3p. The expression level of miR-486-5p constantly increase along erythroid differentiation, while the expression of miR-486-3p reaches a peak at day five and then moderately declines at later stages of differentiation (Figure 1C).

### BCL11A XL is a Direct Target for miR-486-3p

Searching for proteins targeted by either miRNAs we noticed that BCL11A, a negative regulator of HbF expression, is computationally predicted (http://www.microrna.org/microrna, http://www.targetscan.org) to be a putative target of miR-486-3p. Alternative splicing of BCL11A mRNA produce multiple isoforms leading to a minimum of four transcripts (designated XL, L, S, XS) sharing common N-terminus and differing in usage of the 3′ terminal exon and 3′UTR [36]. Only the 3′UTR of the XL variant is predicted to be a possible target of miR-486-3p.

It has been previously shown that primary adult human erythroid cells predominantly express BCL11A XL and L isoforms, whereas embryonic erythroleukemia cell line, fetal liver and primitive erythroblasts express shorter variants and high γ-globin synthesis levels [22]. In order to analyze BCL11A mRNAs expression in our adult PB derived unilineage E cultures we performed Real-Time RT-PCR by using sets of oligonucleotides specifically recognizing the different isoforms (Figure 2A). In line with published results the shorter isoforms were almost undetectable (XS undectable; S poorly detectable), whereas the XL and L variants were expressed during all stages of erythroid differentiation (Figure 2A). Interestingly, the expression level of XL mRNA was particularly higher compared to L isoform (Figure 2A).

Then, we analyzed BCL11A protein expression in unilineage erythroid cultures: at the protein level the XL and L isoforms are indistinguishable due to their similar size [22], [36]. As shown in figure 2B, BCL11A protein is highly and constantly expressed during differentiation/maturation of PB derived erythroid cells, consistent with the low expression of γ-globin chains evaluated in adult erythroblasts (Figure 2C). However, conversely to previously published results [30], [37], in our cell cultures neither BCL11A mRNAs decrease nor major changes in the expression of this protein were detected. These differences may be probably explained by the different experimental context used to analyze BCL11A expression pattern during human erythroid differentiation. We employed a serum-free liquid culture system with Epo and very low doses of IL-3 and GM-CSF, but without Stem Cell Factor (SCF). In particular, the combination of Epo and SCF was shown to increase γ-globin expression influencing growth and differentiation/maturation-time of erythroid cells [32], [33].

The expression pattern of miR-486-3p and BCL11A is consistent with the hypothesis that BCL11A may be a direct target of miR-486-3p. In order to verify whether the XL BCL11A mRNA variant is a direct target of miR-486-3p, we cloned the 3′UTR of the XL-BCL11A isoform downstream of the luciferase reporter gene. Bioinformatic analysis predicted that the seed sequence in miR-486-3p would match XL BCL11A 3′UTR from 1565 to 1572 nucleotides (Figure 2D). The BCL11A 3′UTR construct was co-transfected with either miR-486-3p oligonucleotide or non-targeting oligoribonucleotide (ctr-miR) into the 293T cell line. A 40% repression of the reporter activity was obtained with miR-486-3p, while there was no reduction with the non-targeting ctr-miR. Deletion of the candidate miR-486-3p binding site abrogated miR-486-3p-dependent repression (Figure 2D). Thus, luciferase reporter assay confirmed that the XL BCL11A 3′UTR is a direct target of miR-486-3p.

### Overexpression and Knockdown of miR-486-3p Directly Regulate Endogenous BCL11A Expression and Indirectly Contribute to γ-globin Expression Level

**Figure 3:**
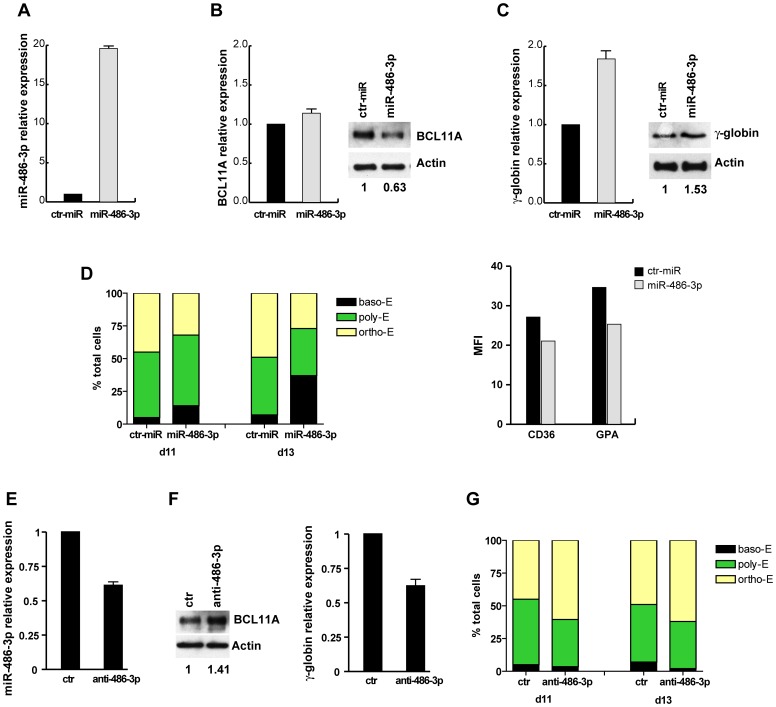
miR-486-3p regulates BCL11A protein levels and affects γ-globin expression. (**A**) Real-Time PCR of miR-486-3p expression in PB unilineage E cultures transfected with miR-486-3p or non-targeting oligonucleotide (ctr-miR). Real-time PCR was performed in triplicate, 48 h after transfection and values are reported as fold changes with respect to ctr-miR. Error bars indicate SD (n = 3). (**B**) *(left)* XL BCL11A expression analyzed by Real-time PCR in 4 days E culture transfected with miR-486-3p or ctr-miR. (*right*) Western blot of BCL11A in transfected E cells seven days post-transfection. Actin was used as loading control. (**C**) The expression of γ-globin was analyzed by Real-time PCR and western blot in transfected cells. Analysis was performed ten days post-transfection (day 11 of culture). Error bars represent and indicate SD from three independent experiments. (**D**) (*left*) Percentage of basophilic (baso-E), polychromatophilic (poly-E) and orthocromatic (ortho-E) erythroblasts ten and twelve days (days 11 and 13 of culture) after transfection. A representative experiment out of three is presented. (*right*) Mean fluorescence intensity (MFI) ratio of CD36 and GPA in transfected E cells analyzed nine days post-transfection. The values are reported as the ratio between MFI of the sample and the isotype control. A representative experiment out of three is shown. (**E**) Real-time PCR of miR-486-3p expression in E cells transfected with anti miR-486-3p LNA (anti-486-3p) or non-targeting LNA oligonucletide (ctr). Analysis was performed three days post-transfection. Error bars represent standard deviation and indicate the average values from three independent experiments. (**F**) (*left*) BCL11A expression analyzed by western blot in E cultures transfected with anti-486-3p or ctr LNA oligonucleotide, 7 days post-transfection. Actin was used as loading control. (*right*) Real-time PCR of γ-globin in transfected cells performed at day 11 of culture. A representative experiment out of three is shown. (**G**) Percentage of differentiating cells at day ten and twelve after transfection (days 11 and 13 of culture).

To investigate the functional role of miR-486-3p on erythropoiesis, we assessed the consequences of its overexpression in erythroid cultures derived from human adult PB HPCs. CD34^+^ cells were grown in unilineage E liquid suspension culture and transfected on day 1 with mimic miR-486-3p, or control oligonucleotide (ctr-miR,). After 48 h the expression level of miR-486-3p in transfected cells was about 20 fold the amount measurable in cells transfected with control oligonucleotide (Figure 3A). The analysis of BCL11A mRNA (XL) and protein expression in E transfected culture was performed 4 and 7 days after transfection, respectively. No fluctuation in BCL11A XL mRNA level was observed in transfected cells (Figure 3B, left). Western Blot analysis showed a decrease of about 40% of BCL11A protein levels in miR-486-3p transfected cells, as compared with the values observed in the control (Figure 3B, right). Consistently, the decreased expression of BCL11A protein was associated to increased expression of γ-globin mRNA and protein, as evaluated by Real-Time PCR and western blot analysis 10 days after transfection (day 11 of culture) (Figure 3C).

We next evaluated whether ectopic expression of miR-486-3p could affect the proliferation and differentiation in E cultures. Erythroid cells transfected with miR-486-3p showed a similar cell proliferation rate, when compared to control oligoribonucleotide-transfected cells (data not shown).

The morphological analysis of erythroid cell differentiation, at days 11 and 13 of culture, showed an increase in the proportion of immature erythroblasts (basophilic erythroblasts) and a concomitant decrease in mature erythroblasts (particularly orthochromatic), in miR-486-3p treated cells compared to control cultures (Figure 3D, left).

We then examined the main immunophenotypical characteristics of E differentiation, such as the expression of the surface glycoproteins CD36 and GPA. Flow-cytometry analysis showed in miR-486-3p E cells a moderate reduction in the percentage of CD36 and GPA positive cells (data not shown), associated with a reduced protein expression at single cell level (MFI) (Figure 3D, right).

To complement this set of experiments, endogenous miR-486-3p was inhibited in erythroid cultures of PB CD34^+^ HPCs by transfecting miR-486-3p-LNA knockdown oligonucleotide or the LNA control sequence. After three days of culture a 40% decrease of the endogenous levels of miR-486-3p was observed in cells transfected with anti-miR oligonucleotide (Figure 3E). Despite the moderate decrease of miR-486-3p after knockdown, higher levels of BCL11A were observed in anti-miR-486-3p transfected cells when compared to LNA control sequence transfected E cells (Figure 3F, left). Accordingly, a decreased expression of γ-globin mRNA was observed 10 days after transfection. (Figure 3F, right).

MiR-486-3p knockdown did not appear to cause marked changes in the differentiation state of the cells, as demonstrated by morphologic analysis (Figure 3G) and by flow cytometry analysis of CD36 and GPA expression (data not shown). These results may be consistent with the relative small changes of miR-486-3p after knockdown of endogenous miR-486-3p.

Altogether, these results indicate that an increased expression of miR-486-3p could contribute to an enhanced expression of HbF, thus suggesting the possibility that altered miRNA 486-3p expression might contribute to different HbF levels observed among β-thalassemia patients.

### miR-486-3p Expression Levels Correlate with Different HbF Content Observed in Unilineage Erythroid Cultures Derived from PB of β-thalassemic Patients

**Figure 4:**
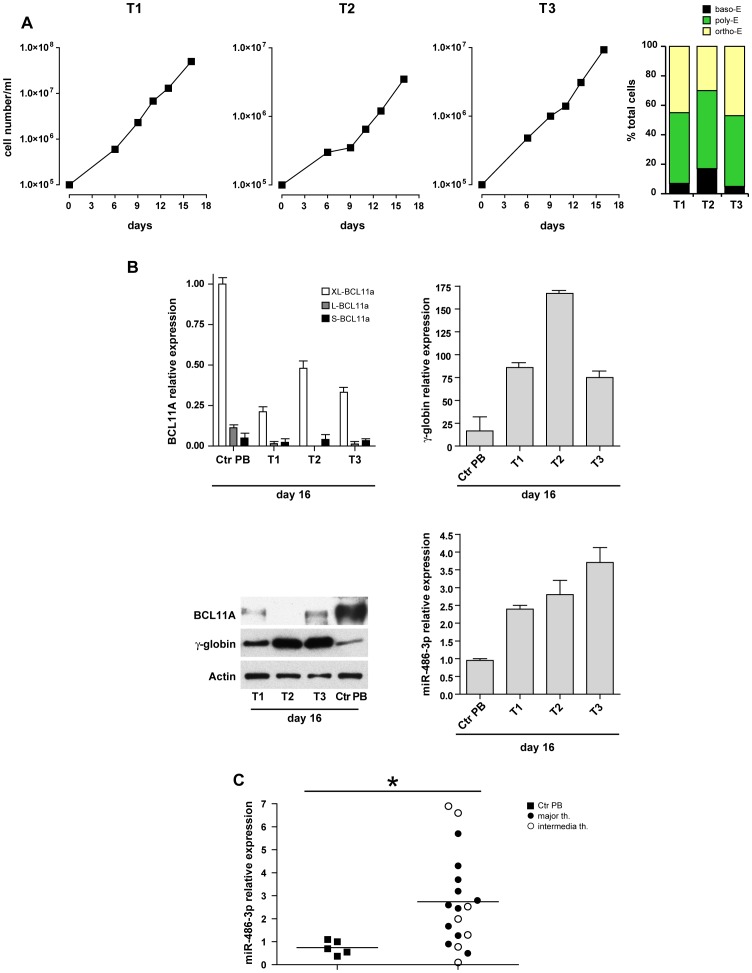
Expression of BCL11A, γ-globin and miR-486-3p in unilineage erythroid cultures derived from thalassemic patients. (**A**) Growth curves and percentage of differentiated erythroid cells in CD34^+^ HPCs unilineage erythroid cultures derived from three β major thalassemic patients (T1, T2, T3). (**B**) Real-Time PCR and western blot analysis of BCL11A and γ-globin in erythroid cells derived from T1, T2, T3 and normal peripheral blood, performed at day 16 of cultures. Actin was used as loading control. Real-Time PCR of miR-486-3p in T1, T2, T3 patients and in normal peripheral blood (day 16 of cultures). (**C**) Real-time PCR analysis of miR-486-3p individual values in erythroid cells derived from peripheral blood of 5 healthy donors and 18 β-major or β-intermedia thalassemic patients.

Then, we analyzed BCL11A levels in unilineage erythroid cultures of CD34^+^ HPCs isolated from peripheral blood of three patients with β-thalassemia major (table 1). The growth curves (Figure 4A) and the percentage of mature erythroblasts (late polychromatophilic+orthochromatic erythroblasts, right panel) were compared with those monitored in normal HPCs culture (Figure 1B). As expected, the growth rate of β-thalassemia major erythroid cultures was lower than that observed in normal subjects and a significant number of cells did not reach terminal erythroid maturation, due to marked dyserythropoiesis. These cultures showed reduced levels of BCL11A mRNAs and protein, associated to a significant increase of γ-globin mRNA and protein expression when compared with their normal counterparts (Figure 4B). Concomitantly, we found that expression of miR-486-3p was significantly increased compared to the levels observed in appropriate controls (Figure 4B).

**Table 1:** Genotype, treatment and clinical course of T1, T2 and T3β-major thalassemic patients.

	Genotype	Treatment	*Clinical Course
T1	β°39/β°39	Transfusion dependent Deferasirox	Hypogonadism
T2	IVSI-1/IVSI-1	Transfusion dependent Desferrioxamine	Diabetes Hypogonadism Osteoporosis
T3	IVSII-745/IVSII-745	Transfusion dependentDeferiprone	HypogonadismHemosiderotic cardiopathy

* Only clinical adverse events are reported.

Then, mature erythroid cells derived from CD34^+^ HPCs cultures of 18 patients affected by major or intermedia β-thalassemia, were analyzed. These cells showed an increase in miR-486-3p expression up to 5–10 fold as compared to levels of miR-486-3p expression observed in erythroid cell cultures from normal PB (Figure 4C). Assuming a cut off of 2 for miR-486-3p expression in normal adult erythroid cells, it appears evident that in 10/18 thalassemic cases miR-486-3p levels are significantly higher than those observed in their normal counterpart. Thus, our data suggest that miR-486-3p expression might contribute to the differentially increased HbF levels observed in thalassemic patients and, possibly, to the clinical severity of the disease.

In order to get rid of the mutual relations between miR-486-3p, BCL11A transcription factor and HbF content, a statistical analysis based on Spearman partial correlation coefficient, which allows to considerate the semiquantitative (rank) character of the data, was computed [38]. The partial correlation allows to check if the correlation between two X and Y variables can be considered as direct or mediated by the intermediation of a third variable Z. Thus, when a relevant direct correlation coefficient between X and Y becomes irrelevant when ‘partialled out’ of the common relation between X and Y with Z, we have a proof-of-concept of the existence of a pathway passing by Z mediating the X and Y relation [38].

The direct Spearman correlation coefficients among the three variables are reported in table 2 together with their *p* values. These coefficients are computed over the entire set of data, each single correlation refers to the subset for which we have the complete information for each couple of variables, the number of couples is reported (N). The results point to a significant relation between HbF content and miR-486-3p (r = 0.775, p<0.002). Further, the negative and significant correlation coefficients between BCL11A and both HbF content and miR-486-3p is also indicated by our data: two consecutive negative relations along an XZY pathway being consistent with a positive correlation between X and Y (table 2).

**Table 2:** Pairwise Spearman correlation between variables.

	HbF content	miR-486-3p	BCL11A
HbF content	1.0 N = 13		
miR-486-3p	0.775 N = 13 (p = 0.0019)	1.0 N = 13	
BCL11A	−0.661 N = 9 (p = 0.052)	−0.797 N = 9 (p = 0.010)	1.0 N = 9

When we computed Spearman correlation coefficient between HbF content and miR-486-3p partialling out the effect of BCL11A, the correlation coefficient dropped from r = 0.775 to a not statistically significant r = 0.566 (p = 0.144), thus indicating the mediation effect of BCL11A in the establishment of miR486-3p-HbF relation. The maintaining of a non statistically significant, albeit non null, correlation between miR-486-3p and HbF, when “partialled out” of the BCL11A effect, indicates the possible existence of an alternative minor pathway linking miR-486-3p and HbF.

On the other hand, the correlation between miR-486-3p and BCL11A, partialling out the effect of HbF content, had a decrease with respect to direct correlation going from r = −0.797 to r = −0.598 (p = 0.117). This result point to a relevant link between miR-486-3p and HbF content and gives a proof-of-concept of the intermediate role played by BCL11A in the relation between miR-486-3p and HbF.

**Figure 5:**
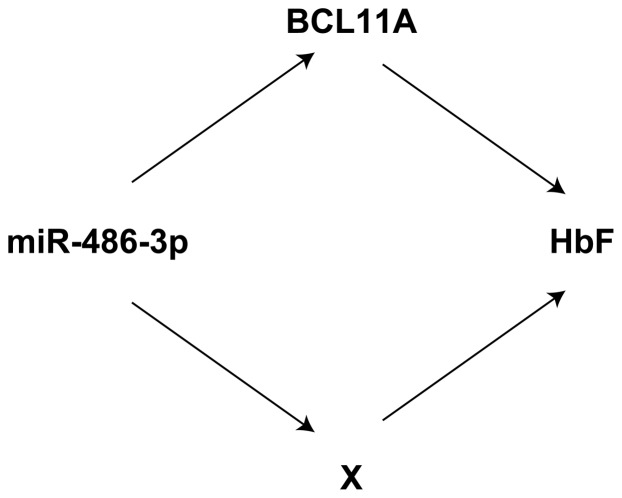
miR-486-3p contributes to regulation of HbF through BCL11A and by BCL11A alternative pathway. A schematic picture of possible pathways linking miR-486-3p and HbF.

These results, with all the caveats coming from the relatively low number of cases, point to a major flux of variation going from miR-486-3p to HbF content through the decrease level of BCL11A protein. Nevertheless, the residual correlation between miR-486-3p and HbF and the substantial invariance of the correlation between BCL11A and HbF content when eliminating the effect of miR-486-3p, points to the existence of alternative BCL11A pathways linking miR-486-3p and HbF content. The general situation arising from our results can be safely described as shown in Figure 5.

## Discussion

In the present study we identified miR-486-3p as a direct inhibitor of BCL11A expression. Our results indicated that miR-486-3p, through regulation of BCL11A expression, may be a critical controller of HbF level.

Genome-wide association studies have identified sequence variants in the gene BCL11A that influence HbF level [39], [40]. Subsequently, it has been demonstrated that BCL11A is a major regulator of HbF switching and critical for the maintenance of γ-globin gene silencing in adult human erythroid cells [22], [23]. The genetic variant within the BCL11A locus, maximally associated with higher levels of HbF, is linked to reduced BCL11A expression. Of note, BCL11A exhibits stage-specific expression: embryonic and fetal cell lines express shorter isoforms, whereas adult erythroblasts express the full-length, XL and L isoforms [22].

Analyzing BCL11A expression pattern in our primary adult human erythroid cells we found that the high molecular weight XL variant was predominantly expressed. We identified XL BCL11A isoform as a direct target of miR-486-3p.

miR-486 is highly conserved among mammals and is transcribed within the *ANK1* locus [41], [42]. The Ank1 gene, encoding an ankyrin repeat domain protein that links the cytoskeleton to the plasma membrane, is transcribed as either a long (erythroid-enriched) [43] or a short (heart muscle- and skeletal muscle-enriched) isoform [44], depending on the cell and tissue types.

No miR-486 sequence has been identified within the genomes of non-mammalian species, such as fishes or birds, although these species have the *Ank1* gene sequence. The human miR-486 precursor generates two mature miRNAs: miR-486-3p and miR-486-5p. MiR-486 is located at Chr:8p11, a region of frequent genomic loss in multiple cancers, and alteration of both mature miRNAs has been linked to cancer [45]. Particularly, miR-486-3p dis-regulation was observed in pancreas and esophageal cancer [46], [47], [48], whereas reduced expression of miR-486-5p was observed in tumoral tissues such as colon, lung, melanoma and gastric cancer [49], [50], [51], [42], [52].

Recent studies identified miR-486-5p as a regulator of PTEN/AKT signaling pathway in cardiac and skeletal muscle. In muscle cells miR-486 seems to be generated by processing intronic RNA from the short ANK1 isoform [41].

In the hematopoietic system miR-486 is primarily and strongly expressed in erythroid cells, but the transcriptional regulatory mechanisms responsible for selective expression within the erythroid lineage remain to be defined. Overexpression of miR-486-3p resulted in a moderate decrease of mature erythroid cells, indicating a possible inhibitory effect on erythropoiesis. These observations suggest that besides BCL11A, dispensable for red cell production and proper maturation of erythroid cells [53], miR486-3p might regulate relevant proteins involved in erythropoiesis.

The observed inhibition of erythroid differentiation may be caused by miR-486-3p repression of proteins regulating erythroid differentiation. One possibility could be that miR-486-3p inhibits erythroid differentiation through regulation of TAL1 (SCL), a transcription factor involved in the regulation of erythroid-specific gene expression program [54]. In fact, bioinformatic analysis indicates TAL1 (SCL) as a potential target of miR-486-3p.

We can not exclude that both mechanisms i.e. the direct control of miR-486-3p on BCL11A expression and miR486-3p inhibition of erythroid differentiation may act together helping each one with the other to decrease BCL11A protein and to increased γ-globin expression.

In this paper we specifically analyzed the role of miR-486-3p as a miRNA involved in the control of γ-globin gene expression by the post-transcriptional regulation of BCL11A expression in differentiating adult human erythroid cells.

This finding raised intriguing questions as to what extent this regulation might contribute to different HbF levels observed among β-thalassemia patients.

Our data on unilineage erythroid cultures of CD34^+^ HPCs isolated from peripheral blood of three patients with β-thalassemia suggest that miR-486-3p expression levels inversely correlate with BCL11A protein levels and directly with HbF content. Moreover, the observed variable levels of miR-486-3p expression in a small cohort of β-thalassemic patients suggest that changes in miR-486-3p expression might contribute to different HbF content observed among β-thalassemia patients.

The presence of a significant molecular pathway involving miR-486-3p - BCL11A - HbF was confirmed by statistical analysis, although all the caveats coming from the relatively low number of analyzed sample have to be considered. Moreover, this analysis also indicates that miR-486-3p might contribute to regulation of HbF expression by using a pathway alternative to BCL11A. This evidence is further supported by computational prediction suggesting the NLI (Ldb1 homolog) protein, involved in negative regulation of γ-globin expression [55], as an additional potential target of miR-486-3p.

In addition, our results suggest that other regulatory mechanisms participate to control BCL11A gene expression. In fact, at the end of maturation period an increased expression of XL BCL11A mRNA associated to unmodified levels of BCL11A protein and to a decreased expression of miR-486-3p, was observed. Thus, it is conceivable that either other miRNAs or regulatory mechanisms diverse from miRNAs may co-operate with miR-486-3p to regulate BCL11A protein expression in erythroid cells.

The possibility that other miRNAs collaborate with miR-486-3p to regulate BCL11A protein expression in erythroid cells can not be excluded. In fact, bioinformatic analysis indicated BCL11A as a potential target of miR-144 which, together with miR-451, specifically regulate erythropoiesis [9], [56]. Further, it has to be considered that BCL11A regulation by miR-486-3p is limited to the XL isoform, the most prominent variant in adult erythroid cells. Therefore, it is conceivable that other miRNAs regulate the BCL11A L isoform, co-operating with miR-486-3p to regulate the overall expression of BCL11A proteins. The concerted action of several miRNAs could lead to a more pronounced decrease of BCL11A and increase of γ-globin expression.

In conclusion, further studies are necessary to more carefully clarify the relevance of miR-486-3p in erythroid differentiation and to identify other targets possibly involved in this process.

Particularly, the mechanisms underlying miR-486-3p overexpression observed in thalassemic erythroid cells remain to be clarified. Further studies into the regulation of miR-486-3p may lead to novel therapies to induce HbF levels in patients with β-hemoglobin disorders, where even modest increases in HbF levels can have a major clinical impact. Moreover, additional analyses are needed to assess the relevance, if any, of these findings in the normal fetal to adult hemoglobin switch occurring during pre- and post- natal development.
